# 

**Published:** 1958-07

**Authors:** 


MEDICAL JOURNAL OF THE SOUTH-WEST

*(Incorporating the Bristol Medico-Chirurgical Journal)*

Established 1883

VOL. 73 (iii) July 1958 NO. 269

### Contents

| | | Page |
|---|---|---|
| EDITORIAL | *C.B.P.* | 55 |
| HOSPITAL CROSS-INFECTION | *Dr. W. A. Gillespie* | 56 |
| ON GROWING OLD | *Dr. J. H. Sheldon* | 69 |
| MEMINGOCOCCAL SEPTICAEMIA WITH BILATERAL ADRENAL HAEMORRHAGE (WATERHOUSE-FRIDERICHSEN SYNDROME) | *Clinical Pathological Conference* | 78 |
| NOTES AND NEWS | | 82 |
| APPOINTMENTS | | 83 |

*PUBLISHED QUARTERLY*

*ANNUAL SUBSCRIPTION 16/- POST FREE*

PUBLISHED UNDER THE AUSPICES OF THE
BRISTOL MEDICO-CHIRURGICAL SOCIETY

### *Editorial Committee*

Dr. J. E. CATES, Department of Medicine, Bristol Royal Infirmary. *Editor.*
Dr. N. J. BROWN, Southmead Hospital, Bristol. *Assistant Editor.*
Dr. J. MACRAE, Ham Green Hospital.
Dr. R. C. WOFINDEN, Central Clinic, Bristol.
Mr. A. E. S. ROBERTS, Medical Library, University of Bristol. *Secretary.*

### *Local Correspondents*

*Bath* . . Mr. A. D. BATEMAN, 3 The Circus, Bath.
*Exeter* . . Dr. L. N. JACKSON, Mount Jocelyn, Crediton, Devon.
*Gloucester* . Dr. R. F. JARRETT, 9 College Green, Gloucester.
*Plymouth* . Dr. C. R. CROFT, Lexden, Hartley Av., Plymouth.
*Taunton* . Dr. M. MCALLEY, 1 The Crescent, Taunton.
*Torquay* . Dr. A. ROBINSON THOMAS, 20 Devon Square, Newton Abbot, Devon.
*Truro* . . Dr. F. D. M. HOCKING, St. Andrews, Perranporth, Cornwall.
*Yeovil* . . Mr. J. A. VERE NICOLL, Knapp House, Yeovil, Somerset.

### NOTICE TO CONTRIBUTORS

Original articles are invited, provided they have not been published elsewhere. Notes of forthcoming events, meetings held, degrees conferred, staff appointments, and other local news are welcomed. The editorial committee reserves the right to make literary corrections.

Illustrations should always be supplied ready for reproduction. All re-touchings or other operations required will be charged to the author. Line drawings should be drawn in Indian ink. We regret that we must ask authors to pay for making blocks, if this is necessary.

J. W. ARROWSMITH LTD., WINTERSTOKE ROAD,
BRISTOL.

### Notes and News

Professor C. Bruce Perry has been appointed Pro-Vice-Chancellor of the University for three years from 1st of August.

Mr. S. D. Loxton has been elected to the Council of the R.C.O.G.

At the inauguration of the new buildings of the Medical Society of the University of Aix-Marseilles the honorary degree of M.D. was accorded to Dr. Macdonald Critchley.

The Queen presented the award of the Polar Medal to Dr. A. F. Rogers for good services as a member of the Commonwealth Trans-Antarctic Expedition.

Mr. P. J. Roylance M.B.BRIST., has been appointed demonstrator in anatomy in the University of Bristol.

Miss Isobel K. E. Brister, M.B.BRIST., D.OBST., has been appointed Assistant M.O. and School M.O., Kirkburton, Denby Dale, Colne Valley, Meltham, Holmfirth and Saddleworth.

Mr. H. S. Paull, M.B.BRIST., D.P.M., has been appointed Specialist (psychiatrist) Northern Nigeria.

A "Bristol Young Doctors' Group" has been formed. Its interests are professional and social. Dr. Derek Zutschi, Southmead Hospital, is Secretary, and anyone interested may obtain further details from him.

Obituary: Dr. J. F. Coates, Bridgwater.

### ARS MEDICA EXHIBITION

A unique collection of medical art by Rembrandt, Dannier, Hogarth, Toulouse-Lantrec and other great masters entitled " Ars Medica ", will be on display in the W. L. Cooper Room in the University Library, 18th to 22nd of August.

### EXAMINATION RESULTS

### *UNIVERSITY OF BRISTOL*

*M.D.*

H. M. Leather.

H. Urich.

*M.Sc.*

Barker, Ruth M.

Khurshid-un-nisa Chaudhri

Mearns, J. T.

*M.B., Ch.B.*

WITH SECOND CLASS HONOURS

Marcus, R. T.
Nicholas, M.
Sharpe, R. A.

PASS

Abraham, R. A. R.

Allen, Daphne M.

Brewer, Sarah M.

Brooking, Barbara A.

Brunning, J.

Bunting, R. A.

Burchill, J. B.

Busson, M.

Coombes, Joan R.

Dada, T. O.

Dent, P.

Egonu, S. V. C.

Entwistle, C. C.

Gardner, P. T.

Huish, R. A. G.

Hulands, G. H.

Lewis, F. W.

Leyton, Susi G.

McConnell, Alison J.

Marot, Yvonne E. J.

Milner, B. S.

Murphy, C. D.

Nagpal, V. P.

Olurin, E. O.

Packer, Glenys R.

Parker, M. L.

Patel, M. P.

Roome, A. P. C. H.

Saunders, M. H.

Seymour, E. J.

Skerrett, F. D.

Slater, J. M.

Surveyor, I.(*with Distinction in Public Health*)

Thean, L. H.

Viswalingam, U.

Ward, J. T.

Whitehead, Juliet M.

Wilmot, Mary G.

*D.P.H.*

Ba Thein

Irani, A. Z.

Lockett, H. I.

Sheerboom, D. J.

Singh, K. M.

Stevenson, Audrey B.

### PRIZES

*Thomas Francis Edgeworth and Francis Henry Edgeworth Prize:* Mr. E. M. Sedgwick.
*Richard Clarke Prize for Child Health:* Mr. R. T. Marcus.
*Edward Fawcett Memorial Prize:* Mr. B. H. Cummins.
*Foyle Prize:* Mr. B. H. Cummins.

### *Appointments*

### *SOUTH WESTERN REGIONAL HOSPITAL BOARD*

### *MARCH—JUNE*, 1958

### *BRISTOL*

| *Name* | *Appointment* | *Formerly* |
|---|---|---|
| SKINNER, P. A., M.B., B.S. (LOND.), F.F.A.R.C.S. | Senior Anaesthetic Registrar, Frenchay Hospital. | Anaesthetic Registrar, Radcliffe Infirmary, Oxford. |
| JOHNSON, W., M.R.C.S., L.R.C.P. | Registrar in Child Psychiatry & Mental Deficiency, Bristol Child Guidance Clinic and Hortham Hospital. | Psychiatric Registrar, St. George's Hospital, Stafford. |
| BURGESS, G. W., M.B., B.S.(SYDNEY). | Surgical Registrar, Weston-super-Mare General Hospital. | Holding same post on locum tenens basis. |

### *NORTH GLOUCESTERSHIRE*

| *Name* | *Appointment* | *Formerly* |
|---|---|---|
| BOREHAM, P. F., M.A., M.B., M.CH.(CANTAB), F.R.C.S.(ENG.). | Consultant Surgeon, North Gloucestershire Clinical Area. | Senior Surgical Registrar, Whittington Hospital, Highgate. |
| MULLALLY, SHEILA, M.B., B.S., D.A. | G.P. Anaesthetist, Gloucestershire Royal Hospital. | Is in general practice in Cheltenham. |
| MCCLELLAND, H. A., M.B., B.S.(DURHAM), D.OBST. R.C.O.G., D.C.H. | Medical Registrar, Cheltenham General Hospital. | Locum appointment at Benshaw General Hospital, Gateshead. |
| GOMPERTZ, R. M. H., M.B., B.S.(LOND.), D. OBST. R.C.O.G., D.C.H. | Clinical Assistant in Paediatrics, Cheltenham General & Maternity Hospitals. | Is in general practice in Cheltenham. |
| HUDSON, JOAN, I., M.B., B.CHIR.(CANTAB), D.P.M. (LOND.). | Assistant Psychiatrist, Horton Road and Coney Hill Hospitals, Gloucester. | J.H.M.O., Fairdene and Netherne Hospitals. |

### *BATH*

| *Name* | *Appointment* | *Formerly* |
|---|---|---|
| JAMES, J. L., B.SC., M.B., CH.B.(B'TOL). | Medical Registrar, Bath Group of Hospitals. | S.H.O. in General Medicine, Bristol Royal Infirmary. |
| BAZELEY, R. W., M.B., B.S.(LOND.). | Clinical Assistants in General Medicine in the Bath Group of Hospitals each undertaking one session weekly. | Is in general practice in Radstock. |
| BURROWES, W. L., M.D., M.R.C.P. | | Is in general practice in Corsham. |
| PINCHING, J., B.M., B.CH. (OXON), M.R.C.P. | | Is in general practice in Wells. |
| RONN, H. H., M.B., B.S. (LOND.), M.R.C.P. | | Is in general practice in Westbury. |
| HIGGINS, P. J., M.B., B.CHIR.(CANTAB). | Clinical Assistant in Paediatrics in the Royal United Hospital, Bath. | Is in general practice in Westbury. |

### DEVON AND CORNWALL

| Name | Appointment | Formerly |
| --- | --- | --- |
| BARTLETT, J. E. A., M.D. (ABERDEEN), M.R.C.P. (EDIN.), D.P.M.(LOND.) | Medical Superintendent, Exminster Hospital, Exeter. | Consultant Psychiatrist, Park Prewett Hospital, Basingstoke; Royal Hampshire County Hospital; Winchester and Aldershot Hospitals. |
| SPENCER, S. J. G., M.A., D.M.(OXON.), D.P.M. (LOND.). | Consultant Psychiatrist, Digby-Wonford Hospital, Exeter. | First Asst. Dept. of Psychological Medicine, University of Durham. |
| SHEACH, JEAN M., M.D., (ABERDEEN), D.M.R.D. | Consultant Radiologist, Exeter Clinical Area. | Assistant Radiologist, United Bristol Hospitals. |
| HEWITT, MARK, M.B., B.S. (LOND.), M.R.C.P. | Consultant Venereologist, West Cornwall Clinical Area. | Also Consultant Dermatologist in West Cornwall Clinical Area. |
| JAIN, C. M., M.B., B.S. (AGRA), D.L.O.(LOND.). | E.N.T. Registrar, South Devon & East Cornwall Hospital, Plymouth. | Out-Patient Officer, Royal National Throat, Nose & Ear Hospital, London. |
| WILKINS, J. L., M.B., B.S. (LOND.). | Paediatric Registrar, South Devon & East Cornwall Hospital, Plymouth (joint appt. with U.B.H.). | Medical Registrar, Essex County Hospital, Colchester. |

### SOUTH SOMERSET

| Name | Appointment | Formerly |
| --- | --- | --- |
| SLIM, A. J., M.R.C.S., L.R.C.P., D.A. | Anaesthetic Registrar to South Somerset Clinical Area based at Taunton & Somerset Hospital. | Anaesthetic Registrar, Royal Bucks Hospital, Aylesbury. |

### UNITED BRISTOL HOSPITALS

### MARCH—JUNE, 1958

| Name | Appointment | Formerly |
| --- | --- | --- |
| COLE, F. L., M.B., B.CH. | Part-time Registrar in Diagnostic Radiology | |
| THOMAS, D. P., M.B., B.S. | Part-time Registrar in Diagnostic Radiology. | |
| MAYO, K. M., M.B., CH.B., D.M.R. | Research Assistant in Radiology. | |

### UNIVERSITY OF BRISTOL

A Short Intensive Refresher Course has been arranged for General Practitioners at Weston-super-Mare Hospital, Weston-super-Mare, from 3rd October to 5th October, 1958

This coincides with the Annual General Meeting of the College of General Practitioners

### *PROGRAMME*

**Friday, 3rd October**

| | | |
|---|---|---|
| 2.30 to 3.30 p.m. | "Micturition Disorders in Children and Adults." | ASHTON MILLER, M.A., M.D., F.R.C.S. |
| 3.30 to 4.30 p.m. | "Vascular Emergencies." | Professor R. MILNES WALKER, M.S., F.R.C.S. |
| 4.30 to 5.00 p.m. | Tea. | |
| 5.00 to 6.00 p.m. | "Recent Developments in Thoracic Surgery." | R. H. BELSEY, M.S., F.R.C.S. |

**Saturday, 4th October**

| | | |
|---|---|---|
| 9.30 to 10.30 a.m. | "Children, their Fears and Problems." | R. F. BARBOUR, M.A., F.R.C.P., D.P.M. |
| 10.30 to 11.30 a.m. | "Diagnosis and Treatment of Thyrotoxicosis." | C. J. FULLER, D.M., F.R.C.P. |
| 11.30 to 11.45 a.m. | Coffee. | |
| 11.45 to 1.00 p.m. | "Domestic Hazards." | A. M. G. CAMPBELL, D.M., F.R.C.P. |
| 1.00 to 2.15 p.m. | Lunch. | |
| 2.30 to 3.30 p.m. | "The Role of Public Health in Geriatrics." | Professor R. C. WOFINDEN, M.D., D.P.H. |
| 3.30 to 4.30 p.m. | "Aural Infections." | H. D. FAIRMAN, M.B., F.R.C.S.E. D.L.O. |
| 4.30 to 5.00 p.m. | Tea. | |
| 5.00 to 6.00 p.m. | "Intestinal Obstruction." | R. V. COOKE, M.S., F.R.C.S. |

**Sunday, 5th October:**

10.00 to 12.45 p.m. Clinical Demonstrations of Cases and Discussion by the Staff of Weston General Hospital.

(Coffee 11.30 to 11.45 p.m.)

Those wishing to attend should apply to:
The Medical Postgraduate Dean,
University of Bristol.

UNIVERSITY OF BRISTOL

The following Sunday Morning Lecture-Demonstrations have been arranged for General Practitioners during the Winter Term 1958

**Sunday, 26th October, Dept. of Med., B.R.I.**
"Management of Sepsis." H. K. BOURNS, F.R.C.S.

**Sunday, 2nd November, Bristol General Hospital**
"Menstrual Irregularities." S. D. LOXTON, F.R.C.S., M.R.C.O.G.

**Sunday, 9th November**
No lecture.

**Sunday, 16th November, Dept. of Med., B.R.I.**
Symposium: "Upper Limb Pain." H. G. MATHER, M.D., M.R.C.P., D. MERVIN JONES, F.R.C.S.

**Sunday, 23rd November, Dept. of Med., B.R.I.**
"Work of the Marriage Guidance Council." H. J. ORR-EWING, M.C., M.D., F.R.C.P., and other speakers.

**Sunday, 30th November, Children's Hospital**
"Affections of the Hip in Childhood." A. L. EYRE-BROOK, M.S., F.R.C.S.

**Sunday, 7th December, Dept. of Med. B.R.I.**
"Regional Ileitis." W. R. BLACK, F.R.C.S.

**Sunday, 14th December, Dept. of Med., B.R.I.**
"Common Dental Emergencies." Professor A. I. DARLING.

Those wishing to attend should apply to:
The Medical Postgraduate Dean,
University of Bristol.

